# Collagen Damage Location in Articular Cartilage Differs if Damage is Caused by Excessive Loading Magnitude or Rate

**DOI:** 10.1007/s10439-018-1986-x

**Published:** 2018-02-08

**Authors:** Lorenza Henao-Murillo, Keita Ito, Corrinus C. van Donkelaar

**Affiliations:** 1 Department of Biomedical Engineering, Eindhoven University of Technology, Gemini-Zuid 4.101, P.O. Box 513, 5600 MB Eindhoven, The Netherlands; 2 Department of Electronics and Industrial Automation, Universidad Autónoma de Manizales, Manizales, Colombia; 3 Department of Orthopaedics, University Medical Center Utrecht, Utrecht, The Netherlands

**Keywords:** Indentation, Mechanical loading, Cartilage damage, Surface roughening

## Abstract

Collagen damage in articular cartilage is considered nearly irreversible and may be an early indication of cartilage degeneration. Surface fibrillation and internal collagen damage may both develop after overloading. This study hypothesizes that damage develops at these different locations, because the distribution of excessive strains varies with loading rate as a consequence of time-dependent cartilage properties. The objective is to explore whether collagen damage could preferentially occur superficially or internally, depending on the magnitude and rate of overloading. Bovine osteochondral plugs were compressed with a 2 mm diameter indenter to 15, 25, 35 and 45 N, and at 5, 60 and 120 mm/min. Surface fibrillation and internal collagen damage were graded by four observers, based on histology and staining of collagen damage. Results show that loading magnitude affects the degree of collagen damage, while loading rate dominates the location of network damage: low rates predominantly damage superficial collagen, while at high rates, internal collagen damage occurs. The proposed explanation for the rate-dependent location is that internal fluid flows govern the time-dependent internal tissue deformation and therewith the location of overstained and damaged areas. This supports the hypothesis that collagen damage development is influenced by the time-dependent material behaviour of cartilage.

## Introduction

Cartilage damage generally progresses into osteoarthritis when adverse biomechanical conditions prevail.^20^,^33^ Among the earliest signs of cartilage degradation are tissue softening^20^ and surface roughening or fibrillation.^15^,^20^ Both are associated with disruption of the collagen network. Because of the slow turnover of collagen in cartilage, collagen damage can be considered an early key indicator of osteoarthritis.^30^ Hence, more insight into the appearance of early collagen damage as a function of mechanical overloading may eventually help to predict the course of the pathology at an earlier stage and improve selection and timing of patient-specific interventions, which is currently challenging.^6^,^11^

Several studies explored the relationship between mechanical overloading and damage to cartilage and its collagen network. Excessive loading magnitude, applied as a single impact or cyclically, results in a series of changes in cartilage, including cell death,^4^,^12^,^16^ softening,^8^,^29^,^33^ loss of network interconnectivity,^28^ fissures^3^ and collagen damage.^2^,^3^,^9^,^29^,^34^ Interestingly, it was also shown that collagen damage may start internally, without visible signs of tissue damage at the surface.^9^,^29^,^34^ At higher loading rates, fissure formation,^5^ proteoglycan depletion,^5^ cell death at different locations^5^,^16^ and micro-cracks occur.^27^ Superficial cracks in the tissue, which also involve collagen damage, have been shown to occur following excessive loading magnitude^2^,^3^ and rate.^5^,^27^ Furthermore, extended duration of loading enhances cell death,^2^,^12^,^13^ proteoglycan loss,^3^,^12^ and collagen damage.^2^,^3^,^12^

Thus, excessive loading magnitude, rate and duration induce a variety of damaging effects in cartilage and result in different appearances of collagen damage. However, the cause of these different appearances is yet unknown. It has been hypothesized that collagen damage results from overstraining of the collagen fibrils. Because cartilage material behaviour is significantly time-dependent due to its biphasic nature, the internal strain distribution will change as a function of loading rate. At fast loading rates, fluid is not given time to flow and tissue deformation is almost isovolumetric. Thus, axial compression results in significant sideways tensile straining of the cartilage and the matrix with its internal collagen network will be strained accordingly. However, under sustained loading or slower loading rates, fluid redistributes and this results in larger compressive straining of the matrix (collagen buckling), but also smaller tensile strains. Accordingly, this study postulates that with the difference in strain distribution, also the locations where tensile strain in the collagen fibers exceeds damage thresholds will be influenced by the loading rate. Thus, it is expected that the location of collagen damage as a consequence of excessive loading at fast rates would differ from these locations at slower loading rates.

The present study aims to build support for the hypothesis that time-dependent tissue behavior affects the appearance of collagen damage, by characterizing the extent and location of collagen damage in articular cartilage as a function of loading magnitude and loading rate. The collagen damage effects will be distinguished in development of superficial clefts and internal collagen network damage. It is hypothesized that similar loading magnitudes applied at higher rates will result in more internal collagen damage as a result of the biphasic, time-dependent mechanical tissue response.

## Materials and Methods

### Osteochondral Plugs

72 osteochondral plugs were harvested from 16 metacarpal proximal epiphyses of 1-year old calves, reaching the skeletal maturity,^1^,^21^ obtained from a local slaughterhouse. Plugs were extracted using a diamond core-drill (Einhell SB 501/1, Einhell, Germany) of 7.5 mm inner diameter and then plugs thickness was reduced to 5–7 mm length with a diamond cut-off wheel (Accutom-5, Struers, Denmark). During drilling and cutting, osteochondral plugs were irrigated with room temperature phosphate buffered saline (PBS) and stored in PBS at – 20 °C until testing.

### Mechanical Testing

**Figure 1:**
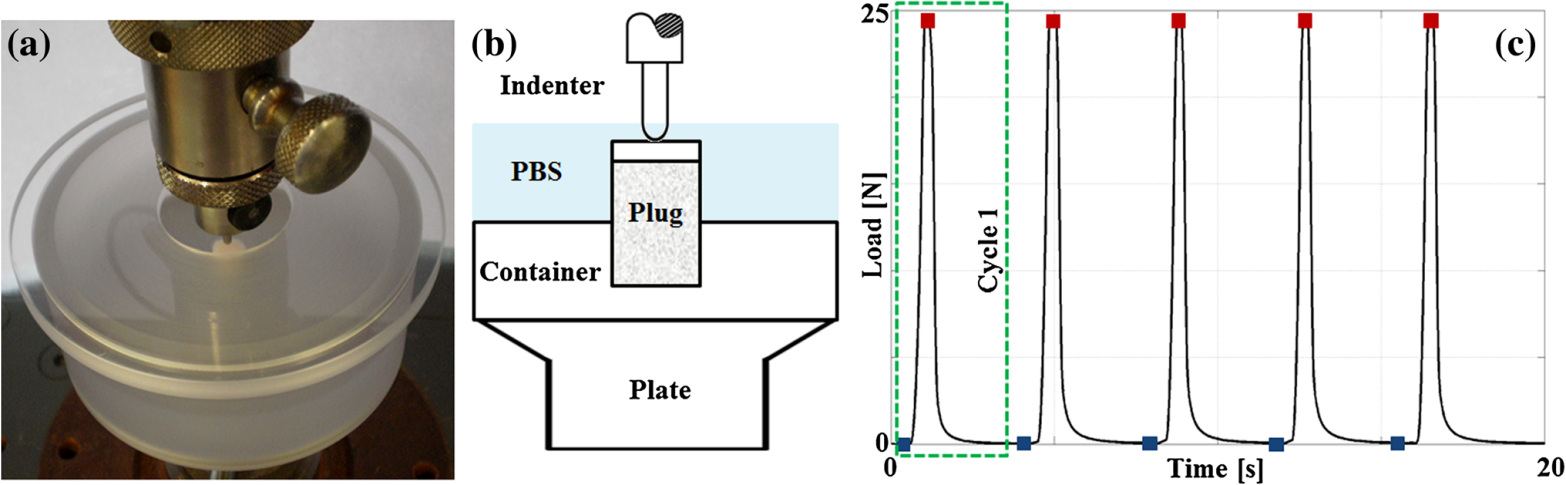
Photograph (a) and schematic (b) of the experimental setup, and the mechanical protocol (c). Indenter reaction force and position were monitored just prior to deformation at rest load (blue squares) and at the peak deformation (red squares) for further analysis.

The plugs were thawed and equilibrated at room temperature for 1 h in PBS. After thawing, cartilage thickness was determined using a digital caliper, by averaging the thickness at four locations along the edge of the cartilage surface. The osseous part of the osteochondral plugs were placed in a custom made polycarbonate container with a 7.5 mm hole in the center of the bottom plate to fix the sample. The container was filled with PBS and covered with a lid to avoid evaporation (Fig. 1a). Samples were slowly press-fit to guarantee the bottom of the samples would be in contact with the container’s hole bottom, by applying a non-damaging compression of 25 N (0.57 MPa at 1 mm/min), distributed over the entire cartilage surface.^31^

To evaluate the local effects of different loading regimes, an impermeable indenter of 2 mm diameter with a hemi-spherical tip (1 mm radius) was used to apply five loading cycles of 15, 25, 35 or 45 N loading at 5, 60 or 120 mm/min, separated by 2.4 s at rest load of 0.05 N (Universal testing machine BT1-FB010TND30, Zwick/Roell, Germany) (Fig. 1). The applied loading magnitudes of 15, 25, 35 and 45 N with the 2 mm hemi-spherical indenter correspond to stresses of approximately 4.8, 8.0, 11.1 and 14.3 MPa respectively. These values are at the high end of physiological loading^19^,^35^ and have been used in other studies for unconfined compression^35^ and indentation.^31^

Immediately after mechanical loading, the cartilage was removed from the subchondral bone with a scalpel and cut into halves. One half was stored in PBS at – 20 °C, the other was embedded in Tissue-Tek compound (Sakura^®^ Finetek, USA, Inc.), rapidly frozen in liquid nitrogen and stored at – 30 °C until histological processing.

### Histology

Cartilage samples were cryo-sectioned in 7 *μ*m slices (Microm HM 550, Germany), mounted on pre-coated glass slides (SuperFrost^®^ Plus, Thermo Scientific, Germany), dried for 1 h and stored at – 30 °C.

For staining, samples were dried for 30 min at room temperature and 1 h at 37 °C, fixed with 3.7% 0.1 M phosphate buffered (pH 7.4) formaldehyde for 5 min, rinsed in PBS and dipped in 0.01% tween PBS. To enhance the permeability of the extracellular matrix, glycosaminoglycans were removed by incubating with 1% hyaluronidase in PBS (Testicular, Type I-s, EC 3.2.1.35, Sigma–Aldrich, US) for 30 min at 37 °C. To block endogenous peroxidase activity, sections were incubated with freshly prepared 1% (v/v) peroxide in absolute ethanol at room temperature for 30 min. Sections were then incubated with 10% normal horse serum (NHS) for 30 min to block nonspecific staining. To visualize collagen damage, slices were incubated overnight at 4 °C with col2-3/4m antibody (1/800) (Mouse monoclonal IgG_1_, Product Nr. 50–1011, IBEX Pharmaceuticals Inc., Canada) in 1% bovine serum albumin (BSA) and rinsed in PBS. Control samples were incubated with 1% BSA. Subsequently, slices were incubated for 1 h at room temperature with biotin-labeled horse anti-mouse antibody (1/400) (IgG (H + L), produced in horse, Vector Laboratories, Inc., USA) and rinsed in PBS. Then, they were incubated with biotin streptavidin detection system (VectaStain Elite ABC, Vector Laboratories, Inc, USA) reagent for 30 min and rinsed 5 min in PBS. Peroxide was detected by incubating with 3′,3′ diaminobenzidine for 7 min and rinsing thoroughly in PBS. Finally, sections were counterstained with Mayer’s hematoxylin, dehydrated and mounted with Entellan^®^ (Merck, Germany). Stained sections were digitized under inverted light microscopy at 5× magnification (Axio Observer, Carl Zeiss, Germany).

### Processing Data and Statistics

Indentation strain was calculated as the ratio between the original cartilage thickness and the displacement of the indenter from the sample surface to the applied loading of either 0.05 N prior to each peak-loading cycle (Fig. 1c, blue squares) or the peak-loading (Fig. 1c, red squares).

The degree of cartilage damage was evaluated using a custom made histological grading system (Table 1). The amount of damage was classified in two categories: macroscopic superficial damage and microscopic internal collagen damage (visualized by col2-3/4m staining). Each category was grouped into five levels ranging from undamaged (score 0) to severely damaged tissue (score 5), similar to the Mankin score.^14^ Four observers received digitized images of the stained sections and an explanation about the scoring system (Table 1). They independently graded macroscopic superficial and microscopic internal collagen damage.

**Table 1:** Histological damage grading system.

Category	Subcategory	Score
Macroscopic superficial damage (irregularities and clefts)	No irregularities	0
	Surface irregularities	1
	Surface damage	2
	Clefts to transitional zone	3
	Clefts to radial zone	4
	Clefts to calcified zone	5
Microscopic internal damage (visualized by col2-3/4m staining)	No staining	0
	¼ of the cartilage thickness	1
	½ of the cartilage thickness	2
	¾ of the cartilage thickness	3
	Full thickness cartilage	4
	No superficial zone staining	+ 0
	Superficial zone staining	+ 1
Total		0–10

The summation of macroscopic superficial and microscopic internal collagen damage was defined as the total collagen damage in cartilage, and was categorized as normal, mild, moderate or severe (Table 2). The grading reliability inter- and intra- observers was assessed with the Kappa coefficient.^26^ Total, macroscopic superficial and microscopic internal collagen damage scores from the 4 observers were averaged per sample.

**Table 2:** Damage degree and its corresponding score range.

Total damage degree	Total damage score (Table 1)
Normal	0–1
Mild	2–3
Moderate	4–6
Severe	7–10

A three-way mixed ANOVA was run to test the effects of loading magnitude and rate on strains within the five loading cycles. Then, a two-way ANOVA was performed to understand the effects of loading magnitude and loading rate on the strains at the 5th loading cycle, and also on total, macroscopic superficial and microscopic internal collagen damage. Outliers were assessed by inspection of a boxplot, normality was assessed using Shapiro–Wilk’s normality test for each cell of the design and homogeneity of variances was assessed by Levene’s test. A square root transformation was performed on strains and on total, macroscopic superficial and microscopic internal collagen damage to accomplish equality of variances. When any of the ANOVA tests showed a significant interaction, an analysis of simple main effects for the corresponding levels was performed with Bonferroni correction. All pairwise comparisons were run for each simple main effect with reported 95% confidence intervals and *p*-values Bonferroni-adjusted within each simple main effect. When there was not a significant interaction, an analysis of the main effect for the corresponding levels was performed. All pairwise comparisons were run where reported 95% confidence intervals and *p*-values were Bonferroni-adjusted. Mean differences were considered significant at the *p* = 0.05 level. A Spearman’s rank correlation was run to assess the relationship between articular cartilage thickness and total, macroscopic superficial and microscopic internal collagen damage. Statistical tests were produced with IBM^®^ SPSS^®^ 23 software, US.

## Results

### Histology

**Figure 2:**
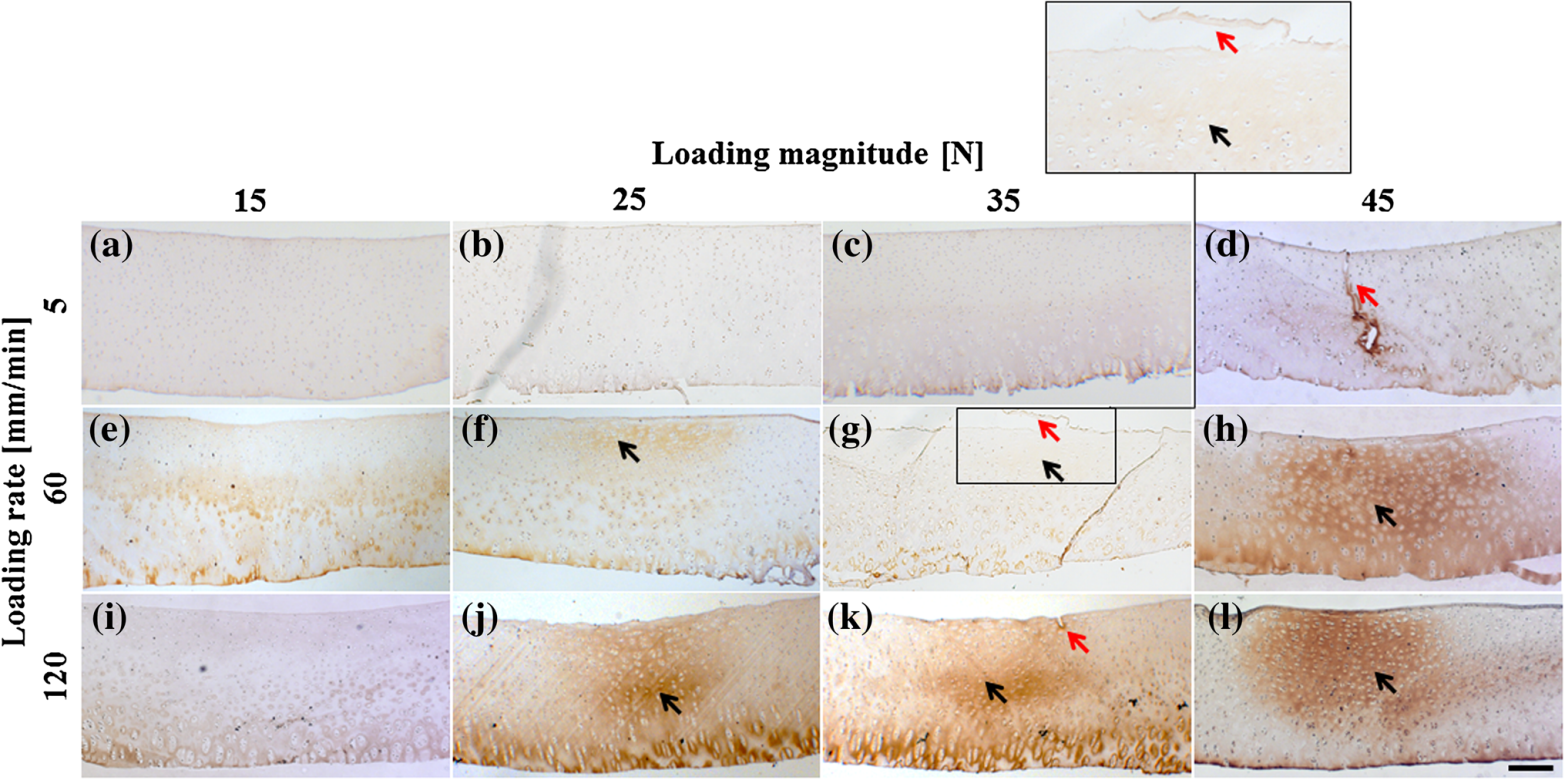
Examples of col2-3/4m staining per group. Brown color indicates denatured collagen and is graded as microscopic internal collagen damage. Note that the brown staining along the attachment to bone (bottom of each image) is natural in healthy cartilage and therefore disregarded in the scoring. Red arrows point at macroscopic superficial damage (g, including insert of damaged area) or fissures (d, k). Black arrows indicate microscopic internal collagen damage areas (f–h and j–l). Images are digitized at 5× magnification; the insert is 20× magnification. Scale bar = 200 *μ*m.

Different collagen damage patterns could be distinguished histologically, depending on the applied loading protocol (Fig. 2). Macroscopic superficial damage occurred in some samples in most groups and was more prominent at higher loading magnitudes (examples indicated by red arrows in Figs. 2d, 2g, and 2k). Microscopic internal damage below the cartilage surface (indicated by black arrows in Fig. 2) occurred predominantly in the groups that received higher loading rates (Figs. 2f, 2j, and 2k) and penetrated to the surface in 45% of the samples (e.g. Figs. 2g, 2h, and 2l). In 13% of the cases, the cartilage surface remained intact, but microscopic internal damage was visible (e.g. Figs. 2f, and 2l).

### Strains

**Figure 3:**
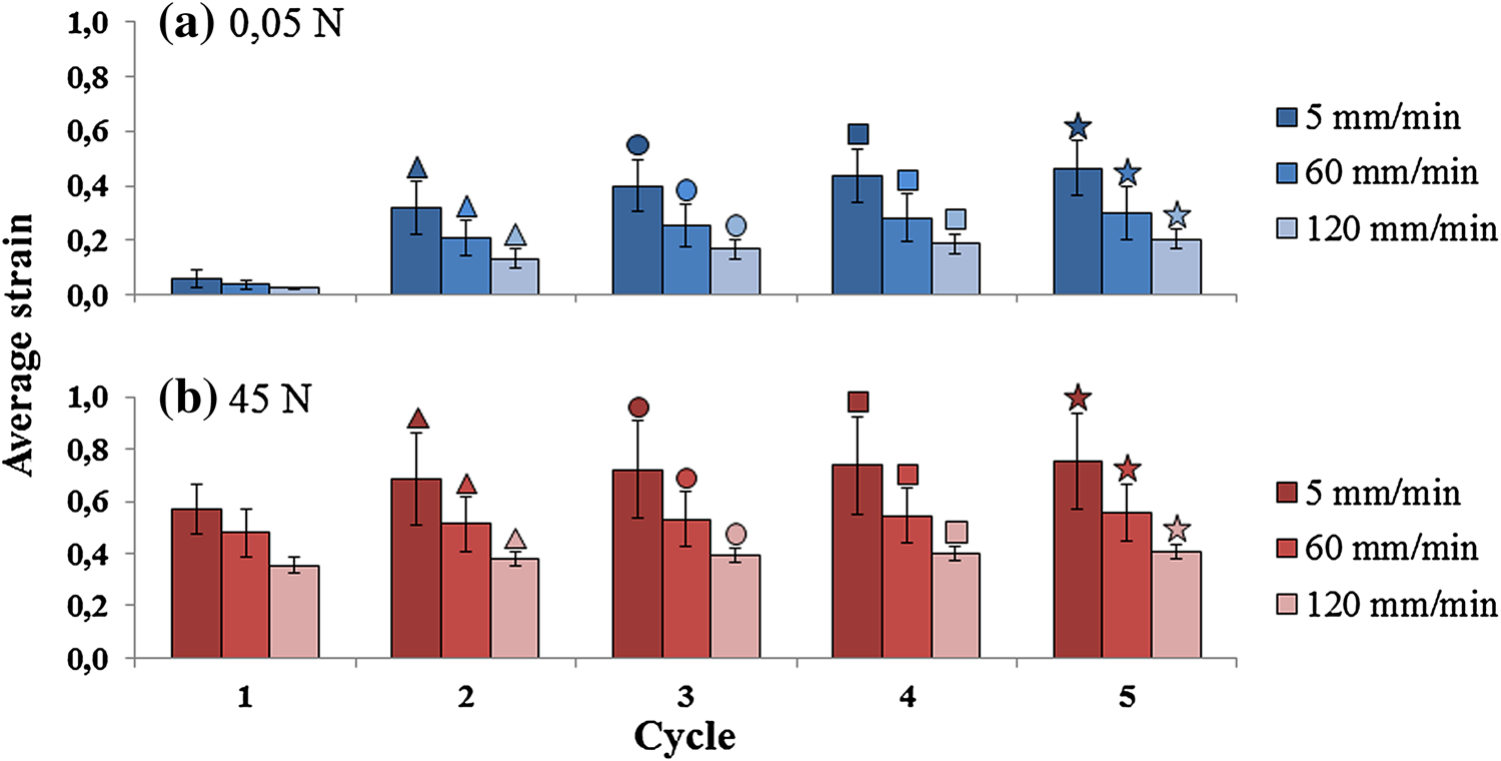
Average strain calculated at every cycle of loading for the group loaded with 45 N. The other groups show the same trend at lower strain values (Fig. 4). (a) Rest load of 0.05 N; comparison within loading rates of 5, 60 and 120 mm/min. The average strain values in rest loading ranges between 0.06 and 0.46 at 5 mm/min; 0.04–0.3 at 60 mm/min; and 0.03–0.21 at 120 mm/min. (b) Peak load of 45 N; comparison within loading rates of 5, 60 and 120 mm/min; *n* = 6 per loading rate group. The average strain values in peak loading ranges between 0.57 and 0.75 at 5 mm/min; 0.48–0.56 at 60 mm/min; and 0.36–0.41 at 120 mm/min. There are statistically significant differences within each cycle of 0.05 N (p < 0.005; blue bars,) and within each peak-loading (p < 0.005; red bars). Every specific symbol (open triangle, open circle, open square, filled star) and color is showing the statistically significant difference between the marked cycle and the previous one. Significance at *p* < 0.05.

**Figure 4:**
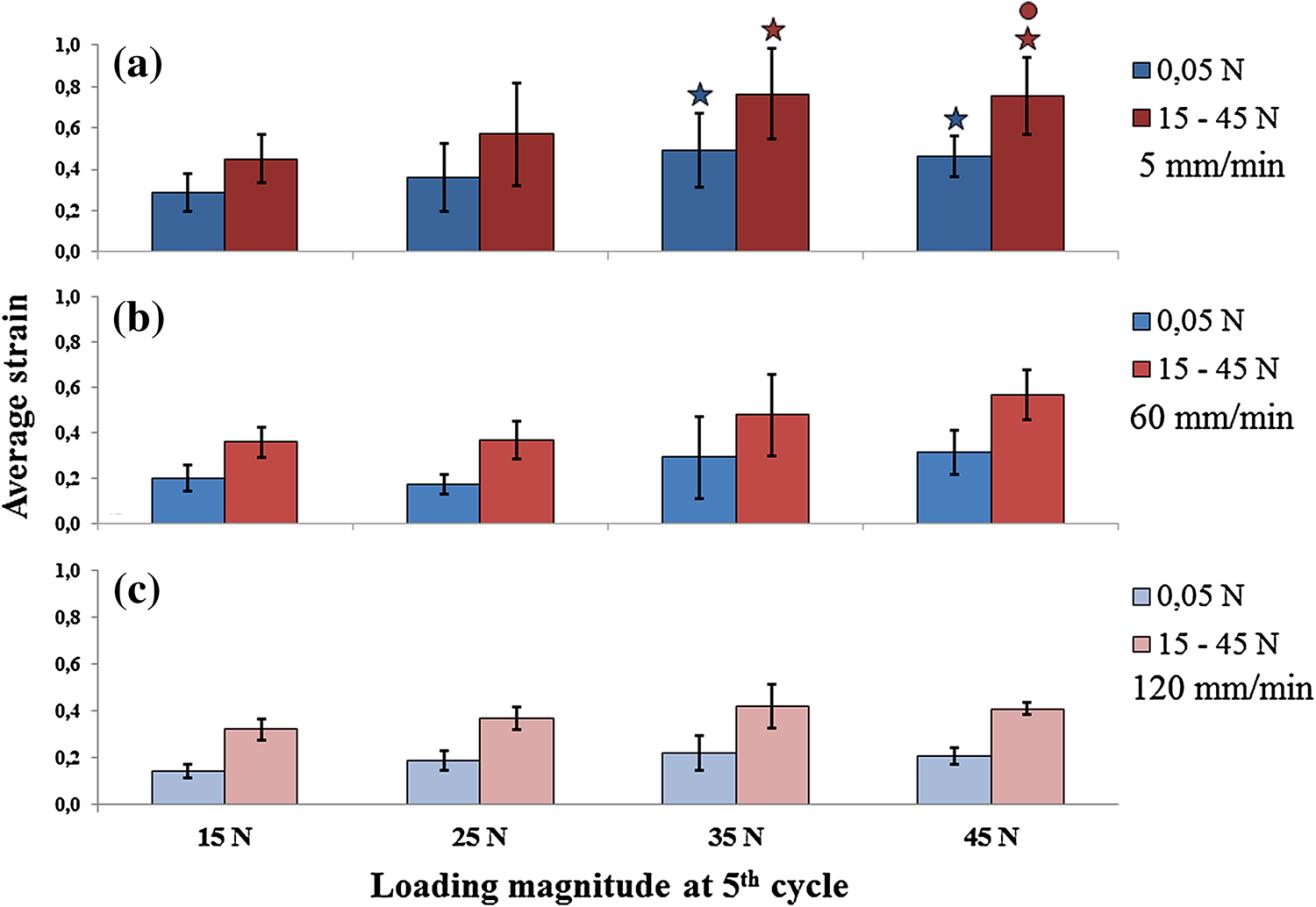
Average strain calculated at 5th cycle of loading for each loading magnitudes applied (horizontal axis, values under bottom graph only). Top: loading rate 5 mm/min. Middle: loading rate 60 mm/min. Bottom: loading rate 120 mm/min. Filled star: statistically significant difference with 15 N; open circle: statistically significant difference with 25 N. Significance at *p* < 0.05.

Average indentation strains increased with each loading cycle, both during the 0.05 N (Fig. 3, blue bars) and the peak-loading period (Fig. 3, red bars). Each new cycle of 0.05 N was statistically significant different compared to the previous cycle of 0.05 N (*p* < 0.005; Fig. 3). This statistically significant difference was also observed within each peak-loading (*p* < 0.005; Fig. 3). The average strains increased when samples were subjected to lower loading rates and higher loading magnitudes. There was no interaction between loading magnitude and rate on strains at 5th loading cycle (*p* > 0.05). There was a statistically significant main effect of loading magnitude at 35 and 45 N, compared to 15 and 25 N (*p* < 0.005; Fig. 4a) and the main effect of loading rate was also statistically significant, comparing 5 mm/min to both 60 and 120 mm/min (*p* < 0.005; Fig. 4a). The increasing strain at 0.05 N loading represents the persistence of an indent in the cartilage, which does not restore to its original height within the 2.4 s of 0.05 N loading. Noteworthy, this indent was still visible in the tissue while the tissue was collected after the loading regime.

### Damage Scoring

The intra- and inter-observer reliability for scoring histological images yielded a weighted (quadratic) Kappa coefficient of 0.9 and 0.69, respectively. This indicated almost perfect intra-observer and good inter-observer concordance strength.^26^

**Figure 5:**
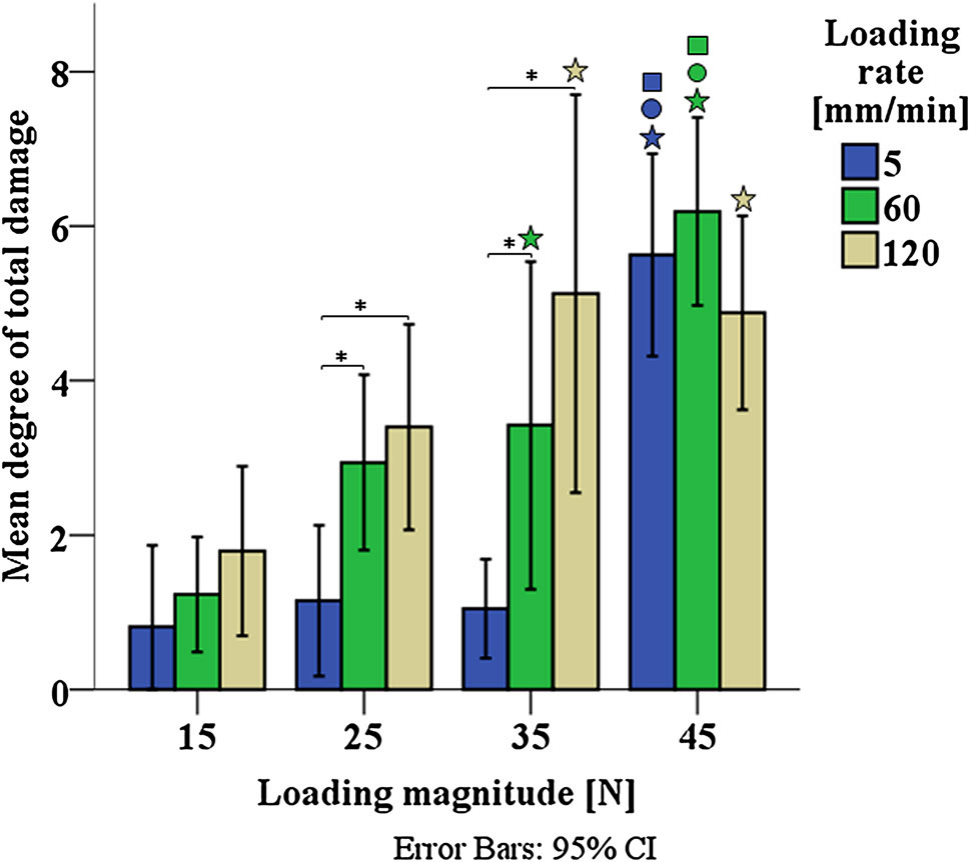
Degree of total damage as a function of loading magnitude and loading rate. Error bars represent 95% confidence intervals. Filled star: statistically significant difference with 15 N; open circle: statistically significant difference with 25 N; open square: statistically significant difference with 35 N; asterisk: statisitically significant difference with 5 mm/min. Filled star, open circle, open square and asterisk: *p* < 0.05.

Few outliers were found by inspecting the boxplots of total, macroscopic superficial and microscopic internal collagen damage; these were kept for being genuine values. According to Shapiro–Wilk’s normality test, most of the damage grades were normally distributed (*p* > 0.05) and Levene’s test showed homogeneity of variances for total (*p* = 0.20), macroscopic superficial (*p* = 0.37) and microscopic internal (*p* = 0.59) collagen damage. Two-way ANOVA showed significant interaction between loading magnitude and loading rate for total collagen damage (*p* = 0.013). There was a statistically significant difference in mean of total damage score between all loading magnitudes (15, 25, 35 and 45 N) at 5 mm/min (*p* < 0.005), 60 mm/min (*p* < 0.005) and 120 mm/min (*p* = 0.016), and between all loading rates (5, 60 and 120 mm/min) at 25 N (*p* = 0.005) and 35 N (*p* < 0.005). Pairwise comparisons for each simple main effect revealed that within each loading rate, loading magnitude had a significant effect on total damage (Fig. 5). In contrast, the effects of loading rate on total damage were only significant in the groups that received intermediate loading (25 and 35 N). Total damage was unaffected by loading rate when loading was 15 N, where all groups showed minor damage, and 45 N, where all groups showed significant damage (Fig. 5).

**Figure 6:**
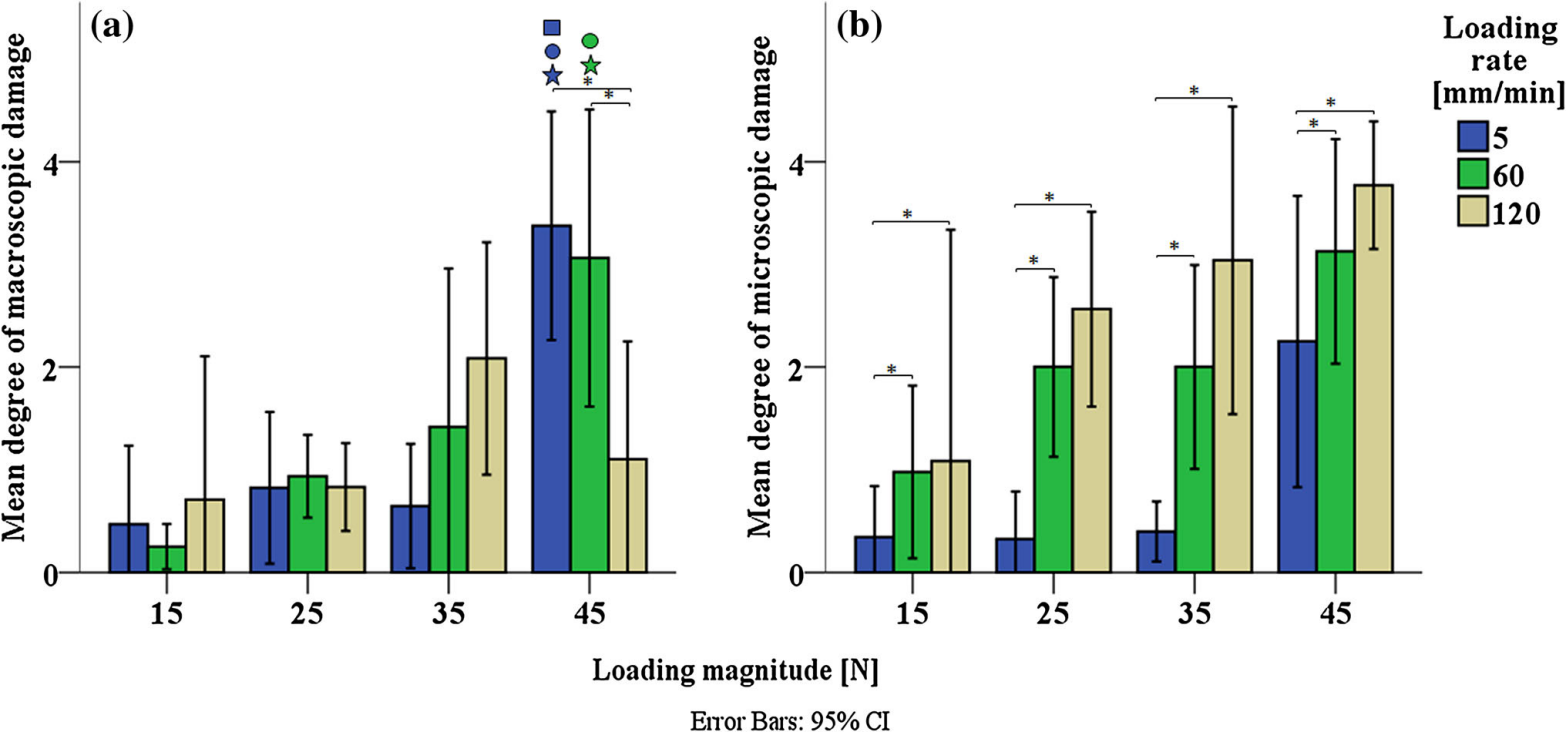
Degree of macroscopic superficial damage (a). Degree of microscopic internal damage (b). Error bars represent 95% confidence intervals. Filled star: statistically significant difference with 15 N; open circle: statistically significant difference with 25 N; open square: statistically significant difference with 35 N; asterisk: statisitically significant difference with 5 mm/min. Filled star, open circle, open square and asterisk: *p* < 0.05.

Two-way ANOVA analysis per type of damage showed significant interaction between loading magnitude and loading rate for macroscopic superficial damage (*p* < 0.005; Fig. 6a), while this interaction did not occur for microscopic internal damage (*p* = 0.23; Fig. 6b). Mean differences of macroscopic superficial damage score revealed a statistically significant difference between all loading magnitudes (15, 25, 35 and 45 N) at 5 mm/min (*p* < 0.005) and 60 mm/min (*p* < 0.005); and between all loading rates (5, 60 and 120 mm/min) at 45 N (*p* < 0.005). Pairwise comparisons for each simple main effect showed that most significant differences in macroscopic superficial damage occurred between 5 and 60 mm/min when a loading of 45 N was applied (Fig. 6a). Microscopic internal damage score indicated a statistically significant main effect of both loading magnitude (*p* < 0.005) and loading rate (*p* < 0.005). Pairwise comparisons evidenced that microscopic internal damage was significant different between 60 and 120 mm/min when loadings of 35 and 45 N were applied (Fig. 6b).

The average cartilage thickness was 1.05 ± 0.23 mm. Spearman’s rank correlation showed a low positive correlation between cartilage thickness and total damage (*ρ* = 0.340, *p* = 0.005), and a moderate correlation between cartilage thickness and microscopic internal damage (*ρ* = 0.436, *p* < 0.005).

## Discussion

The present hypothesis was that time-dependent tissue behavior affects the appearance of collagen damage. The rationale behind this hypothesis was that collagen damage would occur through excessive strains, while the strain distribution in a time-dependent material such as articular cartilage would change with variations in loading magnitude and rate. The approach to test this hypothesis was to assess the location and amount of damage that develops macroscopically in the superficial cartilage layer and microscopically in the internal collagen network, as a function of various combinations of loading magnitudes and loading rates.

In agreement with the hypothesis, results showed that loading magnitude and loading rate are both linked to the degree of total collagen damage in cartilage. Their statistically significant interaction (Fig. 5) suggests variations in appearance depending on the specific combination of loading magnitude and loading rate. Microscopic internal collagen damage increases with both loading magnitude and rate, with no statistical interaction, with suggestions of a loading threshold to induce damage at the highest loading rate and at lower loading magnitudes (Fig. 6b). A different trend is present in the macroscopic superficial damage, which increases with loading magnitude and rate for intermediate loading magnitudes, but with significant interaction; the loading-rate dependency is inverted for the highest loading magnitude (Fig. 6a). Taken together, these data show that different modes of overloading cause distinct appearances of collagen damage in articular cartilage.

Previous studies have demonstrated thresholds of loading magnitude and either loading rate or loading duration at which macroscopic superficial^3^,^5^,^10^,^16^^–^18,24,32 and microscopic internal collagen damage^2^,^29^ develops in articular cartilage. These studies used various experimental setups, including unconfined compression,^5^,^17^,^18^,^24^ drop towers^10^,^32^ or channel indenters.^27^,^28^ The present study used a round indenter to localize the loading. This has the advantage that results are independent of sample size and of the adverse conditions at the cut edges of the sample. Wilson *et al.*^34^ using a similar indenter, found sub-superficial microscopic collagen fiber damage after 25 N indentation, sometimes penetrating to the surface, in agreement with the present results (Figs. 2f–2h, 2j). The same was observed by Chen *et al.*^2^,^3^ under compression with a flat-ended indenter at 5 MPa for 120 min and under confined compression at 1 MPa for 24 h and 5 MPa for 1 h. Unlike other studies, Chen *et al.*^2^,^3^ reported collagen damage to occur only at the surface. However, they used isolated cartilage rather than osteochondral plugs, and attachment to bone is known to affect the mechanical conditions in the cartilage and its collagen network significantly.^10^ Thibault *et al.*^29^ found that stress rates between 2 and 5 MPs/s produce collagen damage, which is mainly concentrated in the deep zone, sometimes extending into the transitional zone.

Macroscopic superficial damage in the present study was mostly limited up to level 3 severity (superficial damage). Only few cases, all at higher loading rates and magnitudes, presented slightly deeper clefts. Full depth fissures may occur as the clefts propagate over longer time, or as a result of more extreme loading magnitudes and rates than those used in the present study.^10^,^32^ The present results concur with other studies which showed that the formation of fissures and cracks increased with loading magnitude^28^ and loading rate,^5^,^17^,^18^,^24^,^27^ with fissures starting from the superficial zone^5^,^17^,^18^,^24^,^27^,^28^,^32^ and propagating into the transitional and deep zone.^10^,^27^

Thus, the general responses at both the microscopic and macroscopic level in the present study were in agreement with previous work. The additional insight from the present study is the differential effect of loading magnitude and rate on the macroscopic superficial and microscopic internal collagen damage.

One possible explanation for this differential effect is that the failure mode of collagen type II may be loading-rate dependent. Strain-rate dependent damage has been reported for fiber-reinforced polymers in general^25^ as well as for collagen type I,^7^ showing that collagen fiber damage started at 10% strain under high strain rates and at 7% strain during quasi-static loading. The present study imposed different strain rates to the tissue surface, but did neither monitor strains nor strain-rates in the tissue in the direction of the local fibers. Therefore, it is not possible to conclude to what extend strain-rate dependent damage of collagen type II could explain the observed effects in this study.

**Figure 7:**
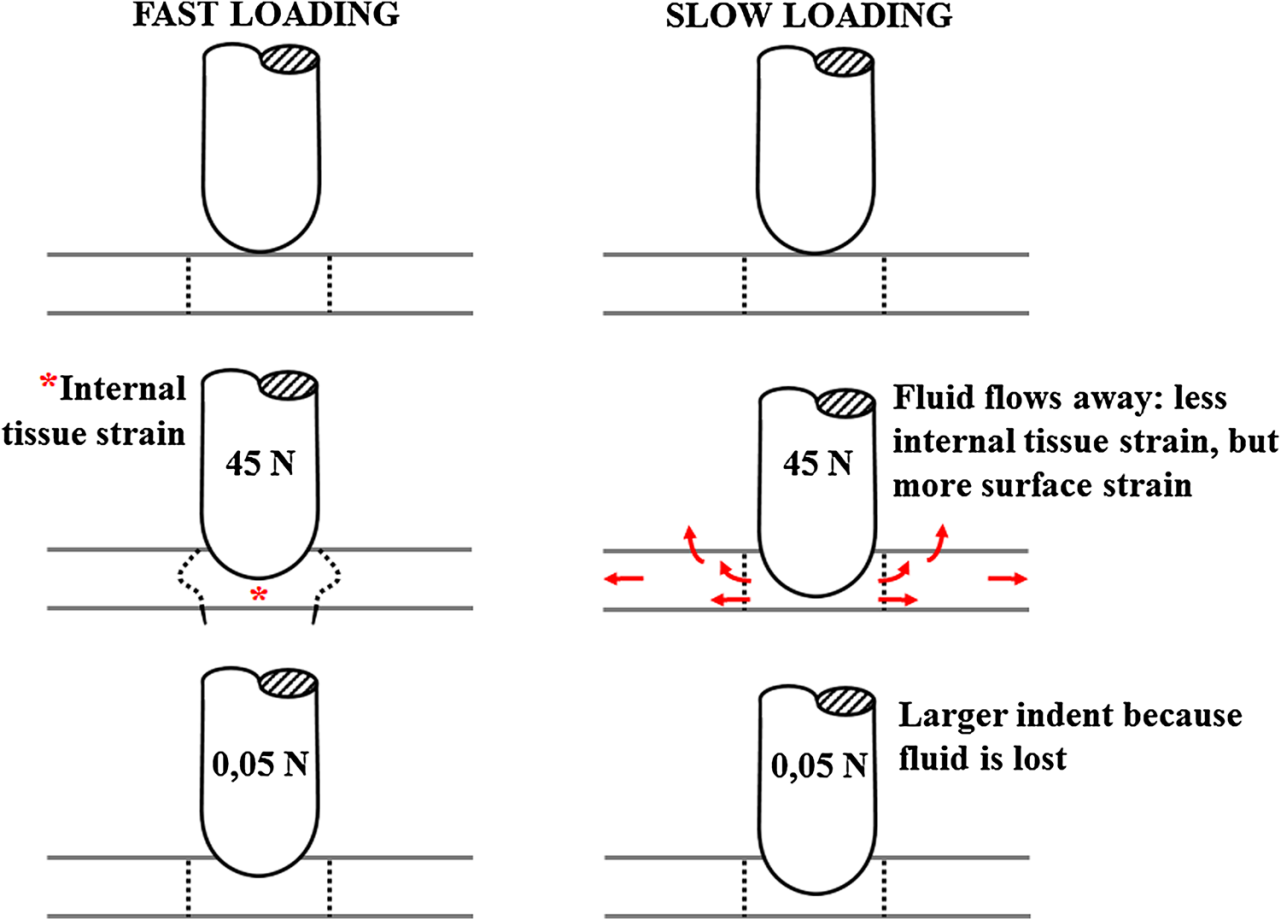
Schematic explanation of the effect of the proposed time-dependent response in the present study. When cartilage is loaded fast (left column), fluid flow is limited and the matrix will deform to accommodate for the imposed shape change (left middle; indicated by *). If the load is released immediately afterwards, total fluid flow remains limited. In contrast, when loading is slow (right column), fluid will have time to flow through the matrix (right middle; arrows indicate fluid flow). The difference in fluid content under the indenter will result in a difference in indentation depth at minimal (0.05 N) loading (bottom row).

The second, more likely explanation for the observed strain-rate dependent collagen damage in cartilage is that the strains experienced by the collagen at the surface and internally in the tissue are influenced in a different way by the biphasic response of the cartilage (illustrated in Fig. 7). During slow indentation, fluid is given time to flow away from the area under the indenter (arrows in Fig. 7), whereas fluid remains in place during fast loading rates. As a consequence of the fluid loss, the average indentation depth increased more for lower loading rates than for higher loading rates. This is evidenced by the indentation depth at the 0.05 N loading period (Figs. 3, 4, 7, bottom). The indent in the cartilage is also visible by the naked eye after the experiment. The deformation of the cartilage surface and its parallel collagen fibers directly follows the imposed deformation by the indenter. Thus, lower indentation rates result in deeper indentations, larger surface stretching and consequently more straining and potentially more damage of the tangential superficial collagen fibers.^22^ This explains the inverse relationship between loading rate and macroscopic superficial collagen damage (Figs. 6a and 7, right column). The same biphasic mechanism results in an opposite time-dependent strain response deeper inside the tissue. At faster loading rates, i.e., at shorter loading durations, fluid is not given time to flow relative to the matrix, and the tissue bulges sideways in the area under the indenter (asterisk in Fig. 7). This causes internal strains in the matrix and in the internal collagen network (Fig. 7, left column). The intermediate zone with its dispersed collagen fiber network is the most susceptible to damage. Thus, at intermediate loading rates and magnitudes, this zone is selectively damaged (Figs. 2f, and 2g), in agreement with former studies. With increasing loading rate (Figs. 2j, and 2k), magnitude (Fig. 2h) or both (Fig. 2l), the damage extends into the deeper zones. Here the staining appears more intense, presumably because the strong anisotropic nature of the tissue in the deeper zone causes a larger number of fibers to become damaged at the same time, when a critical strain threshold in the tissue is crossed.

The above effects are explained by the time allowed for the fluid to move through the tissue. This not only depends on loading rate, but also on local tissue permeability and proteoglycan density, as proteoglycans attract fluid and prevent it from moving. As the effects of fluid flow are dependent on these variables, the depth-dependent cartilage composition as well as variability in cartilage contents between species, joints or locations within joints will influence the quantitative relation between loading rate, matrix straining and collagen damage. Theoretically, the loading rate threshold above which damage may occur in cartilage, will be lower for tissue with lower permeability and higher proteoglycan density. For low or high loading rates, loading rate will overrule the effect of permeability and damage development will occur as presented in Fig. 7.

Recently, strain-rate dependent collagen damage in cartilage was predicted by a computational model.^23^ In a similar range of indentation magnitudes and rates, it was found that collagen damage at the surface would increase when either loading magnitude or loading rate was increased. Also in agreement with the present study is that an interaction between loading rate and magnitude seemed to exist for the internal collagen damage. Only at intermediate loading magnitudes and for intermediate rates, significant collagen damage occurred internally before it became apparent at the surface. However, in the computational predictions it was found that this internal collagen damage would reduce upon further increase in loading rate (Fig. 8 in Párraga Quiroga *et al.*),^23^ whereas it increases in the present experimental data. Thus, the present data provide insight that should be used to improve the damage predictions by the computational model.

The present study used indentation with a 2 mm diameter round-ended indenter. This loading condition was chosen to be in line with former studies,^8^,^23^,^34^ where indentation was chosen as a mechanism to invoke significant collagen strains both at the surface and inside the cartilage. The advantage of indentation is that the result is independent of sample size and of edge-effects due to sample processing. The physiological loading condition where a rounded condyle compresses a rather flat tibia plateau is in between loading with an indenter and unconfined compression. The indenter radius is important for the amount of stress and strain induced in the area below the indenter. Because of the indenter’s round shape, it is difficult to calculate the exact stress level applied to the sample. Presumably, applied stresses reach 5–14 MPa, which is at the high end of physiological loading and comparable to stresses applied by Quinn *et al.* in unconfined compression.^24^

Finally, tissue response varies between samples. Some samples which are subjected to high loading magnitudes and fast loading rates do not show signs of macroscopic superficial or microscopic internal collagen damage, whereas some samples in milder loading groups do. This may be explained by biological variation between metacarpal proximal epiphysis joints of 1 year-old calves. In particular, differences in cartilage thickness have been proposed to affect cartilage damage development.^34^ In the present study a moderate correlation between cartilage thickness and microscopic internal damage was found (*ρ* = 0.436, *p* = 0.0003), suggesting that thickness has an effect but is not the predominant parameter to explain differences between samples. Variations due to the experimental analyses cannot be excluded. For instance, the selected histological slide for evaluation of damage may not be the most central one under the indenter and slices may have different orientations with respect to the split line direction. Such suboptimal histology would result in a slight underestimation of the actual damage. Thus, the presented results can be considered conservative.

To conclude, this study confirms that loading magnitude and loading rate both affect the degree of collagen damage in articular cartilage. It demonstrates that macroscopic superficial damage and microscopic internal collagen damage respond differently to variations in loading magnitude and rate. Macroscopic superficial damage is highest when the indentation depth is largest, i.e., under high loading magnitudes and slow loading rates. Microscopic internal collagen damage occurs when the internal deformation in the cartilage is largest. This internal deformation is governed by the time water is allowed to flow through the matrix. At faster deformations, less water flow can occur relative to the matrix compared to slower deformations. Consequently, faster compression results in more internal straining of the hydrated tissue, and the collagen network is more likely to become overstrained. Damage at the surface is less affected by water displacement, and more dependent on the actual local deformation of the tissue by the indenter. Such differential effects of the loading regime on macroscopic superficial and microscopic internal damage have not been demonstrated before. Further understanding of the mechanically complex, time-dependent mechanisms that result in cartilage damage are important for understanding the etiology and progression of osteoarthritis.

## References

[1] Ahsan T, Harwood F, McGowan KB, Amiel D, Sah RL (2005). Kinetics of collagen crosslinking in adult bovine articular cartilage. Osteoarthr. Cartil..

[2] Chen CT, Bhargava M, Lin PM, Torzilli PA (2003). Time, stress, and location dependent chondrocyte death and collagen damage in cyclically loaded articular cartilage. J. Orthop. Res..

[3] Chen CT, Burton-Wurster N, Lust G, Bank RA, Tekoppele JM (1999). Compositional and metabolic changes in damaged cartilage are peak-stress, stress-rate, and loading-duration dependent. J. Orthop. Res..

[4] de Vries SAH, van Turnhout MC, Oomens CWJ, Erdemir A, Ito K, van Donkelaar CC (2014). Deformation thresholds for chondrocyte death and the protective effect of the pericellular matrix. Tissue Eng. A.

[5] Ewers BJ, Dvoracek-Driksna D, Orth MW, Haut RC (2001). The extent of matrix damage and chondrocyte death in mechanically traumatized articular cartilage explants depends on rate of loading. J. Orthop. Res..

[6] Gardiner BS, Woodhouse FG, Besier TF, Grodzinsky AJ, Lloyd DG, Zhang L, Smith DW (2016). Predicting knee osteoarthritis. Ann. Biomed. Eng..

[7] Haut RC (1983). Age-dependent influence of strain rate on the tensile failure of rat-tail tendon. J. Biomech. Eng..

[8] Hosseini SM, Veldink MB, Ito K, van Donkelaar CC (2013). Is collagen fiber damage the cause of early softening in articular cartilage?. Osteoarthr. Cartil..

[9] Hosseini SM, Wilson W, Ito K, van Donkelaar CC (2014). A numerical model to study mechanically induced initiation and progression of damage in articular cartilage. Osteoarthr. Cartil..

[10] Jeffrey JE, Gregory DW, Aspden RM (1995). Matrix damage and chondrocyte viability following a single impact load on articular cartilage. Arch. Biochem. Biophys..

[11] Kraus VB, Blanco FJ, Englund M, Karsdal MA, Lohmander LS (2015). Call for standardize definitions of osteoarthritis and risk stratification for clinical trials and clinical use. Osteoarthr. Cartil..

[12] Lin PM, Chen CTC, Torzilli PA (2004). Increased stromelysin-1 (MMP-3), proteoglycan degradation (3B3- and 7D4) and collagen damage in cyclically load-injured articular cartilage. Osteoarthr. Cartil..

[13] Lucchinetti E, Adams CS, Horton WE, Torzilli PA (2002). Cartilage viability after repetitive loading: a preliminary report. Osteoarthr. Cartil..

[14] Mankin HJ, Dorfman H, Lippiello L, Zarins A (1971). Biochemical and metabolic abnormalities in articular cartilage from osteo-arthritic human hips. II. Correlation of morphology with biochemical and metabolic data. J. Bone Joint Surg. Am..

[15] Mansour JM, Oatis CA (2009). Biomechanics of cartilage. Kinesiology. The Mechanics and Pathomechanics of Human Movement.

[16] Milentijevic D, Torzilli PA (2005). Influence of stress rate on water loss, matrix deformation and chondrocyte viability in impacted articular cartilage. J. Biomech..

[17] Morel V, Berutto C, Quinn TM (2006). Effects of damage in the articular surface on the cartilage response to injurious compression in vitro. J. Biomech..

[18] Morel V, Quinn TM (2004). Cartilage injury by ramp compression near the gel diffusion rate. J. Orthop. Res..

[19] Morrel KC, Hodge WA, Krebs DE, Mann RW (2005). Corroboration of in vivo cartilage pressures with implications for synovial joint tribology and osteoarthritis causation. Proc. Natl. Acad. Sci. USA.

[20] Mow VC, Gu WY, Chen FH, Mow VC, Huiskes R (2005). Structure and function of articular cartilage and meniscus. Basic Orthopaedic Biomechanics and Mechano-Biology.

[21] Otsuki S, Grogan SP, Miyaki S, Kinoshita M, Asahara H, Lotz MK (2010). Tissue neogenesis and STRO-1 expression in immature and mature articular cartilage. J. Orthop. Res..

[22] Párraga Quiroga JM, Wilson W, Ito K, van Donkelaar CC (2017). Relative contribution of articular cartilage’s constitutive components to load support depending on strain rate. Biomech. Model. Mechanobiol..

[23] Párraga Quiroga JM, Wilson W, Ito K, van Donkelaar CC (2017). The effect of loading rate on the development of early damage in articular cartilage. Biomech. Model. Mechanobiol..

[24] Quinn TM, Allen RG, Schalet BJ, Perumbuli P, Hunziker EB (2001). Matrix and cell injury due to sub-impact loading of adult bovine articular cartilage explants: effects of strain rate and peak stress. J. Orthop. Res..

[25] Ray BC, Rathore D (2015). A review on mechanical behavior of FRP composites at different loading speeds. Crit. Rev. Solid State Mater. Sci..

[26] Sim J, Wright CC (2005). The Kappa statistic in reliability studies: use, interpretation, and sample size requirements. Phys. Ther..

[27] Thambyah A, Zhang G, Kim W, Broom ND (2012). Impact induced failure of cartilage-on-bone following creep loading: a microstructural and fracture mechanics study. J. Mech. Behav. Biomed. Mater..

[28] Thambyah A, Zhao JY, Bevill SL, Broom ND (2012). Macro-, micro- and ultrastructural investigation of how degeneration influences the response of cartilage to loading. J. Mech. Behav. Biomed. Mater..

[29] Thibault M, Poole AR, Buschmann MD (2002). Cyclic compression of cartilage/bone explants in vitro leads to physical weakening, mechanical breakdown of collagen and release of matrix fragments. J. Orthop. Res..

[30] Tiku ML, Madhan B (2016). Preserving the longevity of long-lived type II collagen and its implication for cartilage therapeutics. Ageing Res. Rev..

[31] Torzilli PA, Grigiene R, Borrelli J, Helfet DL (1999). Effect of impact load on articular cartilage: cell metabolism and viability, and matrix water content. J. Biomech. Eng..

[32] Verteramo A, Seedhom B (2007). Effect of a single impact loading on the structure and mechanical properties of articular cartilage. J. Biomech..

[33] Willie BM, Pap T, Perka C, Schmidt CO, Eckstein F, Arampatzis A, Hege H-C, Madry H, Vortkamp A, Duda GN (2015). Overload of joints and its role in osteoarthritis. Towards understanding and preventing progression of primary osteoarthritis. Z. Rheumatol..

[34] Wilson W, van Burken C, van Donkelaar CC, Buma P, van Rietbergen B, Huiskes R (2006). Causes of mechanically induced collagen damage in articular cartilage. J. Orthop. Res..

[35] Zimmerman NB, Smith DG, Pottenger LA, Cooperman DR (1987). Mechanical disruption of human patellar cartilage by repetitive loading in vitro. Clin. Orthop. Relat. Res..

